# Attitudes toward pig islet xenotransplantation for type 1 diabetes: a scoping review

**DOI:** 10.1007/s40618-025-02626-0

**Published:** 2025-06-12

**Authors:** Daniel J. Hurst, Daniel Rodger, Luz A. Padilla, Fernando Ovalle

**Affiliations:** 1 Rowan-Virtua School of Osteopathic Medicine, Department of Medical Education & Scholarship, 40 E. Laurel Road UEC 2135, Stratford, NJ 08084 USA; 2 School of Allied and Community Health, London South Bank University Institute of Health and Social Care, London, UK; 3 Department of Epidemiology, The University of Alabama at Birmingham, Alabama, 35294 USA; 4 Division of Endocrinology, Diabetes, & Metabolism Birmingham, The University of Alabama at Birmingham, Alabama, 35294 USA

**Keywords:** Attitudes, Diabetes, Islet cells, Public perception, Xenotransplantation

## Abstract

**Background:**

Over 8 million people globally have type 1 diabetes. Islet allotransplantation offers an alternative to insulin therapy but is constrained by donor availability. Genetically modified pig islet transplantation presents a potential solution, yet understanding stakeholder attitudes is crucial before clinical adoption.

**Methods:**

This scoping review followed Joanna Briggs Institute methodology and PRISMA guidelines. CINAHL, EMBASE, PubMed, PsychINFO, and SCOPUS were searched for studies examining attitudes toward pig islet xenotransplantation among patients, healthcare workers, and other stakeholders.

**Results:**

From 199 sources, 16 met eligibility criteria, spanning ten countries from 2003 to 2023. The 23,780 participants included 1,535 (6.5%) patients/family members, 216 (0.9%) healthcare workers, and 22,029 (92.6%) students. Attitudes were generally positive but declined sharply in some studies when risks, such as viral transmission, were disclosed.

**Conclusion:**

Despite overall positive attitudes, risk perception significantly influences acceptance of islet xenotransplantation. The predominance of quantitative research highlights a need for qualitative studies and validated survey instruments to enhance understanding and comparability of stakeholder perspectives.

## Introduction

Type 1 diabetes (T1D), an autoimmune disorder affecting over 8 million people globally, results from the destruction of insulin-producing pancreatic β-cells [1]. Treatment typically includes insulin injections and lifestyle modifications. Pancreatic islet allotransplantation is a therapeutic option but is constrained by the limited supply of deceased donor pancreases [2, 3]. Xenotransplantation of islets from genetically modified pigs might provide an additional therapeutic approach for patients with T1D.

Pigs are considered to be a good source of islet cells because pig insulin differs from human insulin by just a single amino acid and has been used successfully in humans for more than 90 years [4]; its use was only discontinued in the US in 2006 [5]. To date, there have been mixed results using porcine islet cells to treat T1D, but some pre-clinical studies have shown promising results, for example, transplantation of porcine neonatal islet cell clusters demonstrated the long-term reversal of diabetes in baboons [6]. The mixed results are best explained by differences and inconsistency with the age of the pigs used (i.e., adult versus neonatal) and the mode of delivery of the islet cells. Human clinical trials are considered to be much less risky than those involving solid organ xenotransplantation, especially where the porcine islet cells are encapsulated as there is no requirement for participants to undertake any immunosuppression therapy [3].

Despite limited clinical testing, understanding public attitudes—including those of patients, family members, and healthcare workers—is essential. Global organizations such as the World Health Organization, International Xenotransplantation Association, and the US Department of Health and Human Services, have all emphasized the importance of public engagement before advancing clinical trials [7–9].

## Methods

A scoping review is a type of evidence synthesis that aims to identify and map the available evidence on a given topic [10, 11]. This scoping review followed the PRISMA extensions for scoping reviews (PRISMA-ScR) checklist, as well as the Joanna Briggs Institute (JBI) methodology for scoping reviews [12–14]. The scoping review protocol was registered on the Open Science Framework registry 10.17605/OSF.IO/7K943.

### Eligibility criteria

To be eligible for inclusion in this scoping review, articles needed to include and evaluate the attitudes of patients, healthcare workers, or other relevant stakeholders such as healthcare and veterinary students specifically toward pig islet xenotransplantation. Articles were excluded if they explored attitudes toward xenotransplantation in general and not islet xenotransplantation specifically. Conference abstracts were included in the search and then excluded if the corresponding full-length peer-reviewed publication was located; if the full-length publication was not located, the abstract was included if it recorded sufficient data and met the other relevant inclusion criteria. Peer-reviewed articles were included if they were published between the period from January 1990 to September 2024 and written in English or Spanish. The initial search year of 1990 was chosen as it corresponds to the first use of fetal pig islet cells in humans, which occurred in a pilot clinical trial by Groth et al. from 1990 to 1993 [15, 16]. Hence, this means that it will capture relevant studies conducted before their first use in humans. The search parameters were chosen to capture articles published from the period when significant advances in xenotransplantation research first occurred. Articles using qualitative, quantitative, and mixed methods are included.

### Search strategy

The following databases were searched to identify relevant literature: CINAHL, EMBASE, PubMed, PsychINFO, and SCOPUS. To be certain that relevant publications would not be overlooked, broad search strings were used combining “islet” and “xenotransplantation” with “attitudes, views, beliefs.” The full search strings that were used for each database can be found in Table 1.

**Table 1:** Database search strings

Database	Search string
CINAHL	“islet“[Title/Abstract] AND “xenotransplantation” AND (“belief” OR “attitude” OR “view” OR “opinion”)
EMBASE	(‘islet’ OR ‘islets’/exp OR ‘islet cell transplantation’/exp) AND (‘xenotransplantation’/exp OR ‘xenograft’ OR ‘porcine islet’) AND (‘belief’ OR ‘beliefs’ OR ‘attitude’ OR ‘attitudes’ OR ‘view’ OR ‘views’ OR ‘opinion’ OR ‘opinions’) AND [embase]/lim
PubMed	“islet“[Title/Abstract] AND “xenotransplantation” AND (“belief” OR “attitude” OR “view” OR “opinion”)
PsychINFO	summary(islet) AND summary(xenotransplantation) AND (“belief” OR “attitude” OR “view” OR “opinion”)
SCOPUS	“islet“[Title/Abstract] AND “xenotransplantation” AND (“belief” OR “attitude” OR “view” OR “opinion”)

### Selection process

**Fig. 1:**
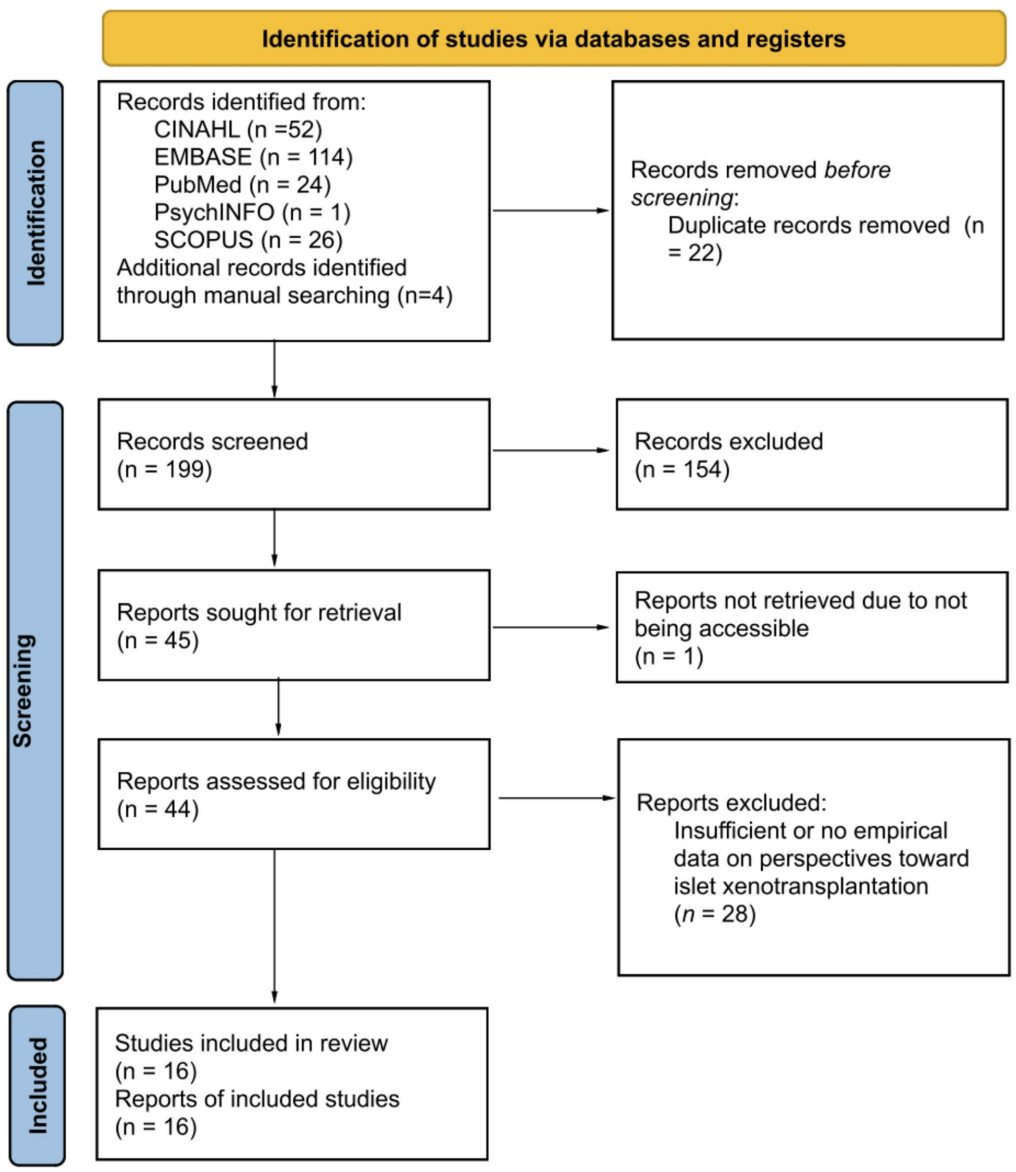
PRISMA-ScR Flow Diagram

The search was completed on October 7, 2024. To help identify additional relevant publications, a manual search was conducted of relevant peer-reviewed transplant journals (e.g., *Xenotransplantation*) and the reference lists of studies identified in the database searches. Records were downloaded into the EndNote citation management software and duplicates were removed. Two investigators first screened the records for eligibility based on the title and abstract. Some records were excluded after this step with agreement by the investigators. Then, the full text of reports of the remaining records were obtained. Two investigators screened the full-text records independently; any disagreement was resolved by team discussion when needed. Figure 1 presents the flowchart of the report selection process.

## Results

### Selection of sources of evidence

After duplicates were removed, this left 199 sources for the initial screening of titles and abstracts. Following this, the full text of 44 studies were screened for inclusion and 16 were retained for inclusion in the review.

### Characteristics of included studies

**Fig. 2:**
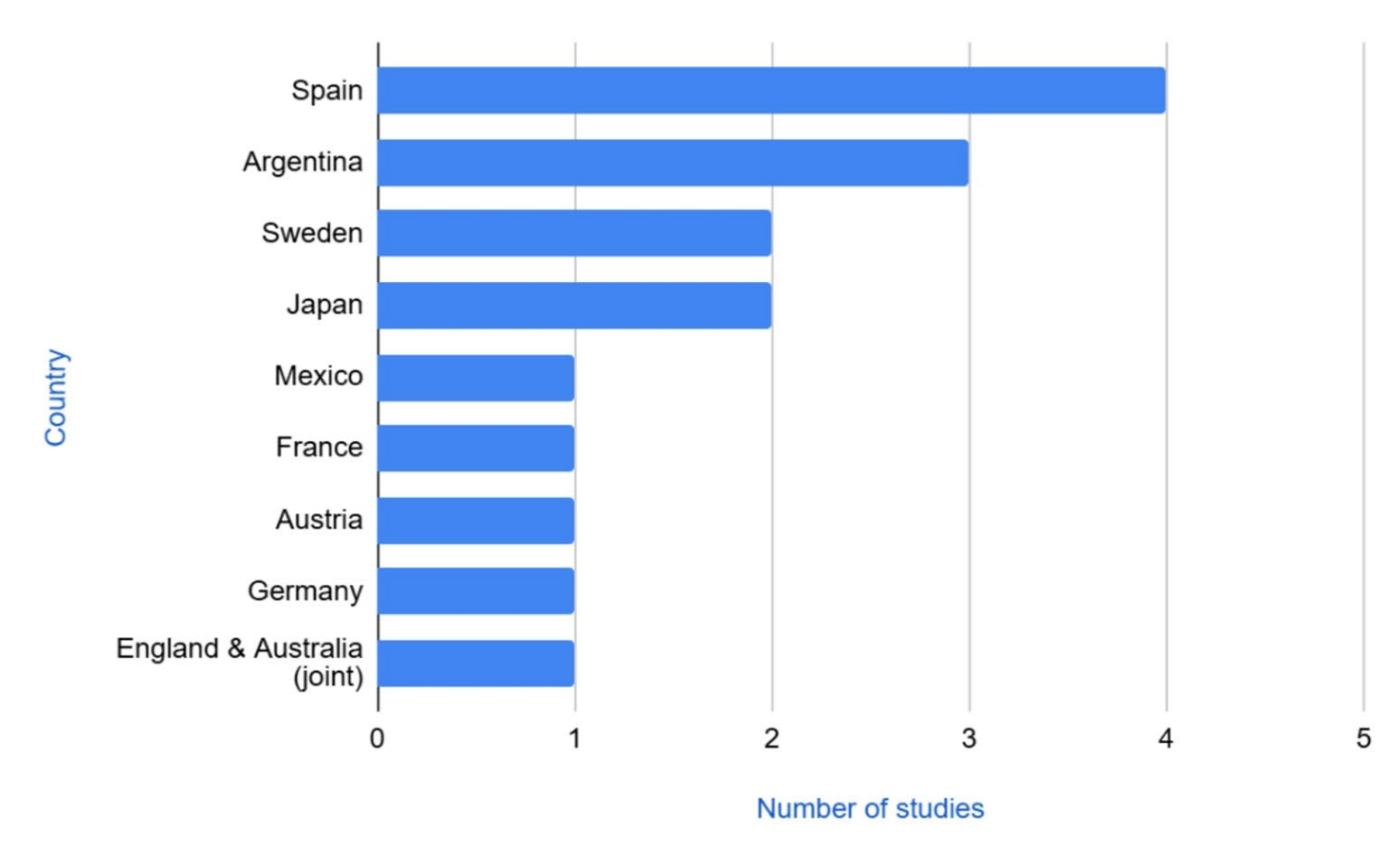
Geographical contributions

The characteristics of the 16 studies included are presented in the data extraction chart (Table 2), and 31% (*n* = 5) of them have been published since 2015. A succinct summary of themes delineated by stakeholder groups is seen in Table 3. The majority of sources were published in the journal *Xenotransplantation* (*n* = 7; 47%). Four studies were conducted in Spain, three in Argentina, two each in Sweden and Japan, with one study being conducted in Mexico, France, Austria, Germany, and one jointly conducted in England and Australia (Fig. 2). Most of the studies used cross-sectional surveys (*n* = 11, 69%) and the remaining studies were either interviews or utilized a mixed methods approach of surveys and interviews (*n* = 5, 31%).

**Table 2:** Included studies

Author(s)	Year	Journal	Country	Population studied	Sample size	Design and methods	Main findings
Idvall and Tibell	2003	*Transplantation Proceedings*	Sweden	Nurses, physicians, researcher, diabetic patients, and family members	*n* = 37	Interviews	Majority of interviewees were positive about the scientific development of xenotransplantation, but ambivalent about the procedure itself.
Deschamps	2005	*Xenotransplantation*	France	Type 1 diabetic patients	*n* = 214	Cross-sectional survey	Initially, 52% indicated they would accept islet xenotransplantation. However, after being informed of possible risks, only around 25% were still agreeable.
Terán-Escandón et al.	2005	*Transplantation Proceedings*	Mexico	Pediatric and adult type 1 diabetic patients who participated in a clinical trial of porcine islet cells, as well as parents of patients	*n* = 10	Interviews and questionnaires	Patient attitudes toward xenotransplantaiton were generally enthusiastic. Parents expressed concerns regarding the newfound autonomy of their children.
Ellison	2006	*Xenotransplantation*	England and Australia	Type 1 diabetic patients in England and Australia	*n* = 511	Cross-sectional surveys (supported by interview)	Majority of respondents would accept islet xenotransplantation to reverse their diabetes. Unknown if the potential risks of xenotransplantation were presented to respondents.
Idvall	2006	*Xenotransplantation*	Sweden	Diabetic patients with renal failure	*n* = 9	Interviews	Interviewees were positive about porcine xenotransplantation, yet were reserved in their estimation of the technology being applied to them. They spoke of its future potential.
Abalovich et al.	2010	*Xenotransplantation*	Argentina	Insulin-dependent and insulin-independent diabetic hospital patients	*n* = 108	Cross-sectional survey	High acceptance of xenotransplantation of porcine islets (79%). The acceptance declined when patients are alerted about the potential risks of viral infection (53% acceptance).
Martínez-Alarcón et al.	2010a	*Transplantation Proceedings*	Spain	Patients on the kidney and liver transplant waiting	*n* = 373	Cross-sectional survey	Majority of both kidney (83%) and liver (85%) patients were in favor of stem cell xenotransplantation to treat diabetes.
Martínez-Alarcón et al.	2010b	*Transplantation Proceedings*	Spain	Veterinary students	*n* = 482	Cross-sectional survey	If xenotransplantation was confirmed as a clinical reality, the majority (97%) of respondents would be prepared to accept cell xenotransplantation. Only a small number of respondents (9%) would be in favor of xenotransplantation if the results were worse than with human donors.
Ríos et al.	2011	*Transplantation Proceedings*	Spain	Secondary school students	*n* = 3,540	Cross-sectional survey	Majority (62%) would accept islet xenotransplantation to treat diabetes. Unknown if the potential risks of xenotransplantation were presented to respondents.
Stadlbauer et al.	2011	*Clinical Transplantation*	Austria	Patients with liver disease, heart disease, kidney disease, and type 1 diabetes who had already received a transplant or were on a waitlist	*n* = 84	Cross-sectional survey	Majority of patients were willing to accept porcine islet cells if it would lead to enahnced quality of life or survival. Acceptance dropped, though, when asked more pointedly if they would accept for themselves.
Shimoda and Matsumoto	2014	*Journal of Diabetes and Metabolism*	Japan	Type 1 diabetic patients and family members	*n* = 85	Cross-sectional survey	Approximately half of patients and three-quarters of family members supported islet xenotransplantation.
Abalovich et al.	2017	*Xenotransplantation*	Argentina	Hospital workers with experience with xenotransplantation clinical trials versus those without	*n* = 196	Cross-sectional survey	Respondents from the hospital with prior clinical trials showed a significantly more positive attitude toward islet xenotransplantation.
Kawabe et al.	2018	*Islets*	Japan	Diabetic patients and family members	*n* = 96	Cross-sectional survey	Xenogeneic islet transplantation was accepted by 66.7% of patients and 88.3% of family members.
Martínez-Alarcón et al.	2019	*Xenotransplantation*	Spain	Healthcare students	*n* = 18,007	Cross-sectional survey	The vast majority (89%) of health science students would accept porcine islet cells if they had type 1 diabetes. 10% of students were unsure about whether they would accept porcine cells and just 1% were not willing. Medical students viewed porcine islet cells slighlt more positively than student nurses.
Kogel et al.	2021	*BMC Medical Ethics*	Germany	Type 1 diabetic patients	*n* = 7	Interviews	Participants viewed porcine xenografts as a last-resort option if standard therapies failed. Participant concerns included perceived discomfort with the procedure, burdensome post-transplant monitoring, and potential career impacts from quarantine due to xenozoonosis.
Matsumoto et al.	2023	*Xenotransplantation*	Argentina	Type 1 diabetic patients	*n* = 21	Cross-sectional survey	The majority (76%) of patients who had recieved an encapsulated porcine islet xenotransplant would do so again and would also recommend it to others. No patients had experienced any psychological issues or been bullied

**Table 3:** Synthesis of results by stakeholder group

Stakeholder Group	Theme/Pattern	Description
Patients and/or Family Members	General Positive Attitude (Initial)	Attitudes were often described as generally positive or even “enthusiastic.” Some noted a “positive but ambivalent” or “reserved” attitude.
	Significant Decline with Risk Disclosure	Acceptance dropped sharply in some studies when potential risks were presented, particularly viral transmission or risks not yet identified.
	Motivations for Acceptance	Key drivers included the desire to be insulin-free, reduce daily dependence on insulin, achieve greater autonomy, improve quality of life or overall survival, and the perceived effectiveness of the treatment.
	Specific Concerns	Burdensome post-transplant monitoring, the impact of potential quarantine on personal/professional life due to xenozoonotic risk, strict follow-up protocols, potential psychological risks associated with living with porcine cells, and concerns for children’s increased autonomy leading to risky behaviors
	Preference for Alternative Therapies	Patients and family members often showed higher acceptance for other emerging therapies like induced pluripotent stem cell therapy and allogeneic islet transplantation compared to xenotransplantation. Xenotransplantation was sometimes viewed as a “last-resort option.”
Healthcare Workers	“Positive but Ambivalent” Attitude	Attitudes were described as generally positive but also “measured in their enthusiasm” due to uncertainty about the clinical application. Attitudes were “positive but ambivalent.”
	Influence of Prior Experience	Attitudes were significantly more positive among staff in hospitals with prior clinical trial experience in xenotransplantation compared to those without.
Students	High Level of Acceptance	Students, particularly healthcare students, displayed a high level of acceptance. This group comprised the vast majority of participants in the included studies. Veterinary students showed exceptionally high acceptance. Medical students were slightly more positive than nursing students
	Factors Correlated with Acceptance	Higher acceptance correlated with a favorable attitude toward deceased organ donation, discussion of transplantation with family and friends, and believing they might need a transplant in the future.

Of the 16 articles included in this review, 11 (69%) studied the attitudes of patients (primarily diabetic patients) and/or family members; one (6%) study assessed attitudes of healthcare workers, and three (19%) studies assessed viewpoints of students. One (6%) study assessed the viewpoints of healthcare workers, diabetic patients, and family members. The total number of participants from the 16 articles was *n =* 23,621. The largest group represented was healthcare students (*n =* 18,489; 78%).

### Patients’ and/or family members’ attitudes

Twelve studies assessed the viewpoints of patients and/or family members toward islet xenotransplantation [17–28]. Of these, three included patients and/or family members (parents) of patients who had received porcine islet cells [17, 19, 28]. First, Idvall and Tibell provided scant details of attitudes, merely concluding that the participants in their study “support the kind of problem orientation that is so fundamental in science” [17]. The researchers defined *problem orientation* as seeing “difficulties as something to be overcome.” Overall, attitudes were described by Idvall and Tibell as “positive but ambivalent.” Secondly, Teran-Escandon and colleagues described the attitude of patients and parents as enthusiastic [19]. Patients (*n* = 10; 14.58 *±* 7.93 years of age) were motivated to participate in the clinical trial in order to (i) reduce their daily dependence on insulin injection, as some expressed that their peers had expressed unpleasant attitudes when they had to inject themselves, (ii) have greater autonomy over their life, and (iii) be able to have food and drinks that would normally be prohibited for diabetics. Researchers described parents’ views on porcine islet xenotransplantation as “ambivalent.” While patients experienced some health benefits, parents found the strict follow-up protocol exhausting, particularly frequent glycemia testing that disrupted sleep. Parents also worried about the unanticipated increase of their child’s autonomy; they feared that this would lead to risky behaviors. The researchers stated that, to the parents, these concerns were confirmed by the start of sexual activity in their children. Thirdly, Matsumoto et al. assessed patients’ (*n =* 21) opinions ten years after receiving encapsulated porcine islet xenotransplantation without immunosuppression [28]. Patients reported improved diabetes control (71%), better blood glucose levels (76%), reduced severe hypoglycemia (86%), and fewer hospitalizations for hyperglycemia (76%). Clinical markers, including HbA1c and insulin dose, also improved. No patients reported cancer or psychological issues. Most (76%) would recommend the treatment, and 85.7% expressed willingness to receive booster transplantation.

Deschamps et al. surveyed 214 type 1 diabetic patients in France about their willingness to undergo pig islet xenotransplantation [18]. Initially, 52% were willing. However, after being presented with possible known risks (e.g., disease transmission) and unknown risks, acceptance dropped precipitously with 74.9% of respondents stating they would refuse islet xenotransplantation. The category of “risks not yet identified” was the greatest concern to 74.2% of respondents, followed by risk of viral infection (36.1%).

Ellison conducted a survey with diabetic patients in England (*n* = 316) and Australia (*n* = 195) [20]. Interviews supplemented some questionnaires in order “to ensure that patients’ views were reflected in their answers;” however it is uncertain from the published data how many interviews were conducted or what questions were asked. Nonetheless, Ellison concluded that most respondents (England, 68%; Australia, 85%) would accept porcine tissue to reverse their diabetes.

Idvall (2006) interviewed nine type 1 diabetic patients in Sweden, including three who previously received islet cell allotransplantation [21]. Despite public discourse framing xenotransplantation as a clinical “breakthrough” (possibly due to the first islet xenotransplantation clinical trial conducted in Sweden by Groth from 1990 to 1993), Idvall noted that his interviewees were positive about the development of xenotransplantation as a clinical therapy, they were also reserved about it. As Idvall noted, “Appearing in the interview narratives is thus a kind of cultural reluctance to believe in the high-soaring imaginations of spearhead science; a reluctance founded on an unwillingness to become overwhelmed by something which obviously still oscillates too much between great promises and great uncertainty” [21]. Effectiveness, rather than the human or animal origin of the transplant, was the key factor for participants.

Abalovich et al. surveyed diabetic patients in Argentina, including 53 insulin-dependent and 55 insulin-independent individuals, about their views on islet xenotransplantation [22]. Overall, 79% (*n* = 86) were accepting of the procedure, with no significant differences based on insulin dependence, complications, or metabolic control. However, acceptance dropped to 57% if viral xenozoonosis risks could not be fully eliminated. Additionally, 40% expressed concerns about potential psychological risks associated with living with porcine cells.

Martínez-Alarcón et al. conducted a cross-sectional study in southeastern Spain with patients on either the kidney (*n* = 214) or liver (*n* = 159) waitlist [23]. As part of the study, participants were asked about animal sourced stem cells for the treatment of diabetes. Among kidney patients, 83% (*n* = 177) supported the use of animal stem cells, 10% (*n* = 22) were undecided, and 7% (*n* = 15) opposed. Similarly, 85% (*n =* 134) of liver patients were in favor, 14% (*n =* 22) undecided, and 1% opposed (*n =* 2). No significant differences were found between the two groups regarding their attitudes toward stem cell xenotransplantation.

Stadlbauer et al. surveyed patients at an Austrian transplantation outpatient clinic, including transplant recipients (*n* = 81%) and waitlist patients (*n* = 13.1%; others did not respond to this question) [24]. Patients primarily visited the clinic due to liver disease (52.4%), heart disease (41.7%), kidney disease (15.5%), and diabetes (20.2%) (multiple answers possible). Twenty-seven patients (32.2%) had diabetes. Most patients (91.7%) were generally willing to accept porcine pancreatic islet cells if it would lead to enhanced quality of life or overall survival. Similarly, 82.1% were accepting of encapsulated porcine pancreatic islet cells to treat diabetes, but only if all risks and side effects could be excluded. When asking more pointedly whether participants would accept islet xenotransplantation for themselves, only about 50% of participants as a whole stated they were willing, whereas 66.7% of participants with diabetes said that they would.

Two studies examined attitudes toward porcine islet xenotransplantation in Japan among T1D patients and their families. Shimoda and Matsumoto analyzed 85 questionnaires, including 53 from patients and 32 from family members, exploring opinions on four emerging therapies: allogenic islet transplantation; islet xenotransplantation; DNA vaccination; induced pluripotent stem cell therapy [29]. Over one-quarter of patients were dissatisfied with current treatments, and 92.5% wanted to be insulin-free. Over half of patients (52.2%) and most relatives (78.3%) supported islet xenotransplantation for themselves or their family members. Acceptance toward islet xenotransplantation ranked second for family members and third for patients, following allogenic islet transplantation. Of the four emerging therapies, patients (77.3%) and family members (96.2%) showed the most acceptance toward induced pluripotent stem cell therapy. Reasons for these attitudes were not assessed.

Kawabe, Matsumoto, and Shimoda conducted a questionnaire-based study in Japan with T1D patients (*n* = 47) and their family members (*n* = 49), assessing viewpoints toward four emerging therapies: single and multiple allogeneic islet transplantation, single and multiple encapsulated allogeneic islet transplantation, single and multiple xenogeneic islet transplantation, and induced pluripotent stem cell therapy [26]. Over 90% of respondents accepted at least one therapy, with family members showing greater acceptance overall. The most favorable treatment for both patients and their family members was induced pluripotent stem cell therapy (83.3% and 95.6%, respectively) followed by xenogeneic islet transplantation (66.7% and 88.3%, respectively) and multiple xenogeneic islet transplantation (46.3% and 71.4%, respectively).

Kögel et al. conducted semi-structured interviews with seven T1D patients in Germany to explore their informational needs for consenting to islet xenotransplantation [27]. Participants viewed porcine xenografts as a last-resort option if standard therapies failed. If the conventional therapies continued to meet their needs, participants did not see any reason to change. They emphasized the importance of involving diabetes and transplant specialists in the process and prioritized quality of life. Concerns included perceived discomfort with the procedure, burdensome post-transplant monitoring, and potential career impacts from quarantine due to xenozoonosis. Participants also stressed the need for financial and medical safety nets in case of treatment failure. None expressed ethical concerns about using animals in xenotransplantation. Notably, this was the only interview-based study on this topic published since 2006.

### Healthcare workers’ attitudes

Two studies—conducted in Sweden and Argentina approximately 15 years apart–with a total of 216 healthcare workers, explored attitudes toward islet xenotransplantation [17, 30]. Healthcare workers included nurses, physicians, researchers, laboratory technicians, hospital administrators and undefined clinical staff members. Idvall and Tibell conducted “free interviews” with healthcare workers in Sweden, noting that while participants were positive about the scientific development of islet xenotransplantation, they were measured in their enthusiasm due to uncertainty about its clinical application. The researchers described the attitudes as “positive but ambivalent,” though, specific details on viewpoints were lacking. It is not clear what questions the interviewees were asked. “Free interviews” may be synonymous with unstructured interviewing, which is an interview methodology in which questions are not predetermined but rather rely on spontaneous conversation.

Abalovich et al. (2017) surveyed clinical staff in two hospitals in Buenos Aires, Argentina [30]. One of the hospitals had performed clinical trials of islet xenotransplantation and the other had not. All respondents were asked three questions (two specific to islet xenotransplantation) regarding their views about themselves or a close relative and their willingness to accept islet xenotransplantation if it could improve or cure their diabetes. Respondents from the hospital with prior clinical trials showed a significantly more positive attitude toward islet xenotransplantation, and the researchers posited that more information about the benefits and risk-safety profile of xenotransplantation would likely increase acceptance among healthcare workers.

### Students’ attitudes

Three studies with a total of 22,029 participants assessed the viewpoints of students toward islet xenotransplantation [31–33]. Veterinary students, high school students, and healthcare students’ viewpoints were assessed. Martínez-Alarcón et al. (2010) assessed viewpoints of veterinary students (*n* = 482) in Spain toward organ, tissue, and cell xenotransplantation [31]. Assuming that xenotransplantation became a clinical reality, 97% (*n* = 467) were prepared to receive cell xenotransplantation.

Rios and colleagues utilized a validated survey to assess the viewpoints of secondary school students in Spain (*n* = 3,540) regarding their acceptance of xenotransplantation for the treatment of diabetes [32]. A majority of students (62%; *n* = 2,195) responded that they would accept cells of animal origin if they suffered from diabetes, whereas 28% (*n =* 1,005) were uncertain, and 10% (*n* = 340) were against xenotransplantation.

Martínez-Alarcón et al. (2019) surveyed 18,007 medical and nursing students, finding that 89% (*n* = 15,972) would accept islet cell xenotransplantation if they suffered diabetes, while 10% (*n* = 1789) were unsure, and 1% (*n* = 246) were opposed [33]. There was no statistical significance between attitude toward xenotransplantation and academic year, sex, or age. Medical students displayed slightly more positive attitudes than nursing students. There was a high level of acceptance of islet cell xenotransplantation among those students who (i) had a favorable attitude toward deceased organ donation (*P* < 0.000), (ii) had discussions of transplantation with their family (*P* < 0.001) and friends (*P* < 0.001), and (iii) had a belief that one might need a transplant in the future (*P* < 0.001).

## Discussion

From 1990 to 1993, Carl-Gustov Groth performed the first-in-human studies of porcine fetal islet transplantation [15, 16]. Significant improvement in glycemic control was not achieved in these studies [3]. Since then, limited porcine islet transplantation has occurred, though recent advances in genetic modification and immunosuppressive regimens have provided renewed excitement about the potential for this novel therapy [34].

In general, many of the participants in the studies we reviewed expressed positive attitudes toward the idea of islet cell xenotransplantation. However, in at least two studies, acceptance declined sharply when participants were presented with risks such as transmission of viruses; similar reactions have been found in the solid organ xenotransplantation literature [35].

The vast majority of the studies identified relied on quantitative methodologies, highlighting a significant gap in the literature regarding qualitative approaches. After 2006, a total of 11 studies were identified for review. Among these, only one employed qualitative methods, emphasizing the underrepresentation of in-depth, context-specific explorations of the subject matter. This lack of qualitative research is particularly notable given its potential to provide rich, nuanced insights into participants’ lived experiences, perceptions, and the complexities of the topic. Consequently, this disparity underscores the need for further qualitative investigations to complement the predominance of quantitative studies and to achieve a more holistic understanding of the field.

There was significant overlap concerning the attitudes toward islet cell xenotransplantation and solid organ xenotransplantation. This can be observed in similar patterns among responses from specific groups; for example, medical students viewed islet cell xenotransplantation more positively than nursing students, matching what was found toward solid organ xenotransplantation [35]. It is unclear why this is the case; it could be that medical students are more likely to already be aware of xenotransplantation or to have interacted more closely with transplant teams and patients during their training.

People living with T1D have also raised concerns about how burdensome the post-transplant monitoring requirements might be, as well as the impact of quarantine due to xenozoonosis on their personal and professional lives. These issues have been discussed in the literature primarily in the context of solid organ xenotransplantation [36]; however, the same principles and problems remain relevant. Any post-transplant monitoring and surveillance requirements should be as minimally burdensome as necessary to ensure the safety of the recipient and wider population. Importantly, informed consent cannot guarantee that a recipient will commit to continue to comply with any post-transplant requirements and there is no legal precedent to mandate it. The infection risks can be more nuanced because, where the porcine islet cells are encapsulated the risk of xenozoonosis may be lower [37]. Nevertheless, the absolute risk of xenozoonosis is very low, and even lower for the wider population but never zero [38].

There was considerable methodological heterogeneity of the included studies, particularly concerning measurement tools and reporting standards. Many of the studies, especially earlier investigations, reported limited details regarding their methodologies, and only a single study utilized a validated questionnaire. This reliance on non-validated instruments and often sparse reporting of methodological details presents a considerable impediment to the advance of knowledge in this field. Such methodological inconsistency severely limits the comparability of findings across different studies. Without standardized and validated assessment tools, it is challenging to accurately aggregate data, replicate results, or confidently draw generalized conclusions about attitudes toward porcine islet xenotransplantation, thus hindering the accumulation of a cohesive and reliable evidence base. This lack of comparability ultimately impedes the ability to synthesize findings definitively or to identify consistent patterns or effects that could inform clinical practice or regulation.

Moving forward, these limitations underscore critical directions for future research. To enhance the rigor and comparability of studies there is a clear and urgent need for the development and validation of standardized instruments. This would allow for more precise, consistent, and comparable measurements across diverse research settings and participant populations. There is also the need for the increased adoption of methodological designs that include mixed methods approaches. While quantitative studies are vital for measuring outcomes of large populations, incorporating qualitative insights can provide a more comprehensive and nuanced understanding of complex phenomena, addressing both *what* is happening and *why* in real-world contexts. Lastly, longitudinal approaches may be considered. Given the often evolving nature of viewpoints toward novel medical therapies, longitudinal studies are helpful tools to track changes over time, which cross-sectional studies cannot fully capture.This scoping review has the following limitations. First, while the researchers used broad search strings in several databases, followed by manually searching references, it is still possible that some relevant articles may have been missed. Second, our objective was to describe the reported viewpoints toward islet xenotransplantation, and, therefore, did not explore the different variables that could influence viewpoints. Third, researchers used disparate means to assess attitudes. While some studies reported detailed viewpoints, others were brief in their results, simply reporting on one dependent variable (attitude toward islet xenotransplantation). In addition, the methodology and data analysis used by researchers was oftentimes not reported in detail. Fourth, the majority of viewpoint data (> 90%) comes from students—including those in secondary school and in various health sciences. This may limit generalizability to a patient population.

## Conclusion

In summary, this scoping review reveals that most participants in the included studies displayed an overall positive attitude toward islet cell xenotransplantation for the purpose of treating diabetes. However, a notable caveat found in certain studies was that after being informed of certain risks, attitudes shifted from overall positive to a negative attitude. Importantly, a consistent limitation across the body of literature was the methodological variability: several studies reported limited details regarding their methods, only four were qualitative studies, and only one utilized a validated questionnaire. Hence, while the existing quantitative data provides a valuable overview of attitudes toward islet cell xenotransplantation for treating diabetes, its generalizability and comparability are currently limited by the methodologies employed. Based on these findings, we identify several specific and actionable research needs to advance this field: (1) development and validation of standardized instruments; (2) expansion of qualitative research; (3) inclusion of diverse patient groups to capture different perspectives from those individuals who may one day have pig islet xenotransplantation as a clinical option. Addressing these specific research needs will be critical for building a more complete, nuanced, and actionable evidence base to inform patient education, policy and regulatory guidance development, and the ethical integration of islet cell xenotransplantation as a potential therapeutic option for diabetes.
